# Multigear Bubble Propulsion of Transient Micromotors

**DOI:** 10.34133/2020/7823615

**Published:** 2020-02-21

**Authors:** Amir Nourhani, Emil Karshalev, Fernando Soto, Joseph Wang

**Affiliations:** ^1^Department of Nanoengineering, University of California San Diego, La Jolla, CA 92093, USA; ^2^Department of Mechanical Engineering, Department of Biology, Biomimicry Research and Innovation Center, University of Akron, Akron, OH 44325, USA

## Abstract

Transient, chemically powered micromotors are promising biocompatible engines for microrobots. We propose a framework to investigate in detail the dynamics and the underlying mechanisms of bubble propulsion for transient chemically powered micromotors. Our observations on the variations of the micromotor active material and geometry over its lifetime, from initial activation to the final inactive state, indicate different bubble growth and ejection mechanisms that occur stochastically, resulting in time-varying micromotor velocity. We identify three processes of bubble growth and ejection, and in analogy with macroscopic multigear machines, we call each process a gear. Gear 1 refers to bubbles that grow on the micromotor surface before detachment while in Gear 2 bubbles hop out of the micromotor. Gear 3 is similar in nature to Gear 2, but the bubbles are too small to contribute to micromotor motion. We study the characteristics of these gears in terms of bubble size and ejection time, and how they contribute to micromotor displacement. The ability to tailor the shell polarity and hence the bubble growth and ejection and the surrounding fluid flow is demonstrated. Such understanding of the complex multigear bubble propulsion of transient chemical micromotors should guide their future design principles and serve for fine tuning the performance of these micromotors.

## 1. Introduction

Micro/nanoscale motors, capable of efficient propulsion and complex operation, are at the forefront of research in micro- and nanotechnology and robotics [1]. Bubble-propelled chemically powered autonomous micromotors—based on different designs, e.g., rockets or spheres—have been developed over the past decade to perform diverse tasks in biomedical, environmental, and industrial applications [2–8]. Additionally, external fields such as magnetic field have been employed to successfully guide and control the motion of micromotors [9]. The engines in the early generations of bubble-propelled micromotors employed catalytic degradation of fuels, such as hydrogen peroxide and sodium borohydride by materials such as platinum, palladium, or manganese oxide [10–14]. While these micromotors offered proof of concept for microscale self-locomotion, their widespread use is restricted by the incompatibility of their fuels with biological environments. Another obstacle was the retrieval of the micromotor, made of nondegradable materials, upon completion of its task.

Transient, chemically powered micromotors—a new microengine generation—address these challenges. In an attempt to move away from toxic fuels, expensive catalysts, and nondegradable leftovers, a push towards biofriendly materials has begun in recent years in the micromotor community [15, 16]. To incorporate biodegradability for *in vitro* and *in vivo* applications, these micromotors are powered by the consumption of active metals, such as magnesium (Mg), zinc, and iron, which react with biofluids or seawater [17–22]. These micromotors self-propel *via* single replacement reactions in gastric acid, or *via* reaction with water in intestinal fluid, salty buffer solutions, or basic buffer solutions where the counter ions aid in removing the passivating byproduct layer of magnesium hydroxide [16].

The ability of Mg-based micromotors to propel in biological fluids with minimal risk has enabled their use for important applications, such as drug delivery, where micromotors outperform passive diffusion-based methods [17–20, 23]. To fine tune the performance of these micromotors and provide design principles, it is crucial to understand their dynamics and the underlying mechanisms of motion. Unfortunately, the transient nature of these Mg-based micromotors and the variation of their active material over their lifetime make them a more complicated system to study compared to chemically propelled hollow shell micromotors with constant active area [24–27]. Moreover, while a hollow tubular microrocket has an opening for fluid entrance and another opening for bubble ejection [28–30], these Mg-based transient micromotors have only one opening that serves for both purposes. Additionally, time-dependent depletion of the active metal inside a transient micromotor increases the complexity of the bubble-propulsion mechanisms. Therefore, current theoretical models for bubble-propelled catalytic micromotors cannot address the dynamics of transient micromotors, and a new framework is needed to understand their distinct time-varying propulsion behavior.

In this paper, we investigate the mechanisms of bubble propulsion for transient chemically powered micromotors and identify the elements of micromotors' powered motion and stochastic dynamics. We investigate self-locomotion over the lifetime of micromotors and analyze the different processes involved in their motion. Our study identifies distinct patterns in the formation and ejection of bubbles, calling each pattern a *gear*, in analogy with macroscopic multigear machines. We also investigate the distinct behavior emerging from the effect of hydrophobic and hydrophilic shell surfaces upon the bubble propulsion and motion of such transient micromotors. Finally, we discuss the fluid flow around the stationary and motile microengines. We hope that our multigear framework described here, along with the new understanding of the bubble growth and ejection and influence of the shell polarity, could give insight into the future design and engineering of a wide range of high-performance transient chemical micromotors.

## 2. Results and Discussion

**Figure 1:**
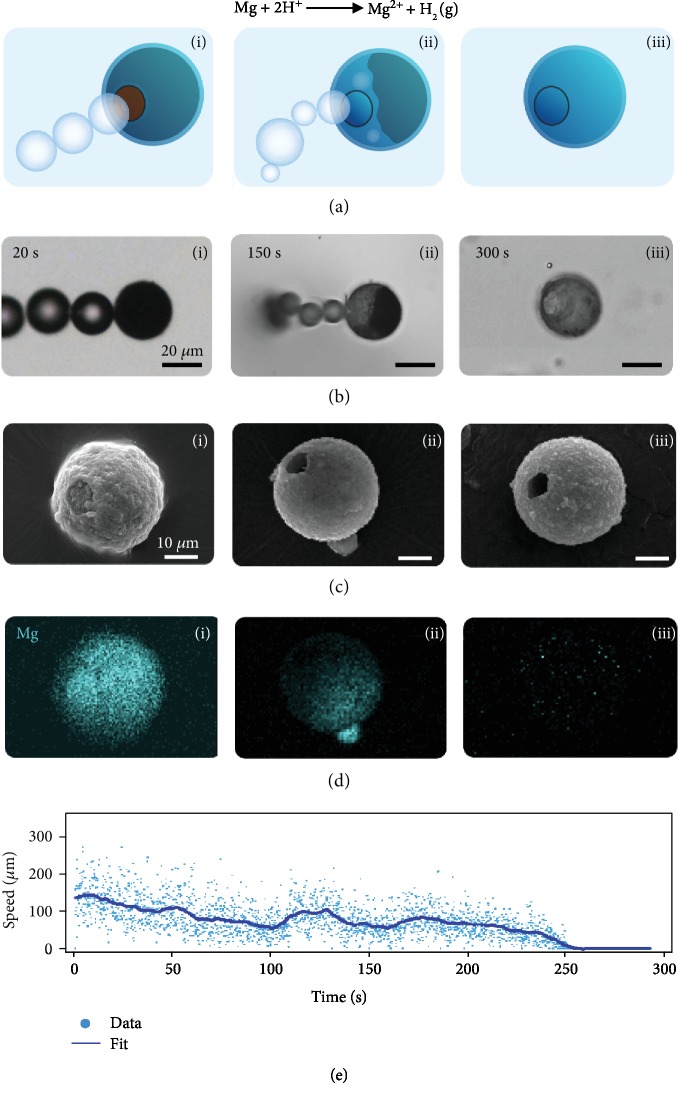
Time-dependent changes in the internal structure and speed of a Mg-TiO_2_ micromotor. (a) Schematic, (b) micrograph, (c) SEM image, and (d) accompanying EDX Mg signal maps of micromotors at different times during their lifetime, from beginning (i), middle (ii), and end (iii). (e) Instantaneous speed of a Mg-TiO_2_ micromotor during its lifetime until complete Mg depletion.

We studied the behavior of Mg-based transient micromotor with hydrophilic (titanium dioxide) and hydrophobic (parylene) insulating shells [31]. A typical micromotor is fabricated by coating an Mg microparticle (diameter 20-25 *μ*m) with an insulating material using atomic layer deposition (Fig. [Supplementary-material supplementary-material-1]). The thickness of a titanium dioxide (TiO_2_) shell is ~170 nm for the majority of our analysis, and the thickness of parylene is ~500 nm. One area of the Mg particle (facing the substrate) is not covered by the insulating material and serves as an “opening,” through which Mg is exposed to the reactive solution, in our case simulated gastric acid (pH ~1-2). The reaction of Mg with the acid results in the production of hydrogen molecules, leading to nucleation, growth, and ejection of bubbles (Figure 1(a)). Micrographs of a typical Mg-TiO_2_ micromotor propelling in simulated gastric acid illustrate the bubble production and parallel depletion of the Mg core over time (Figure 1(b)). Scanning electron microscopy (SEM) (Figure 1(c)) and energy-dispersive X-ray spectroscopy (EDX) (Figure 1(d)) images at different stages of micromotor lifetime demonstrate such gradual depletion of the Mg core over time. Throughout this depletion, the speed of the micromotor undergoes a large variation. Figure 1(e) shows a typical micromotor's instantaneous speed (dots) alongside a local average fit (solid line). [Supplementary-material supplementary-material-1] demonstrates an alternative version of Figure 1(e) with a jagged line representing the speed profile of the micromotor. The fluctuations in the speed suggest that random processes and stochastic dynamics are involved in the micromotor dynamics during its lifetime, until complete Mg depletion and, hence, the stop of micromotor motion at ~250 s. To understand the underlying mechanism behind these phenomena, we study the dynamics of these micromotors at shorter time scales.

**Figure 2:**
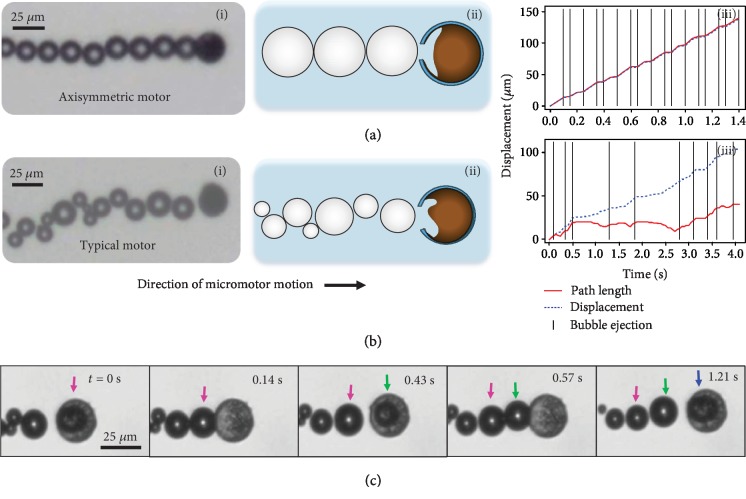
Observed propulsion mechanisms of Mg-TiO_2_ micromotors. (i) Micrograph, (ii) schematic illustration, and (iii) the path length and net displacement versus time. Vertical lines in (iii) represent the appearance of a new bubble for (a) an axisymmetric and (b) a typical Mg-TiO_2_ micromotor. (c) Sudden fluid jet mechanism for propulsion upon bubble nucleation inside a micromotor. Colored arrows track sequential bubbles in order of ejection.

Figure 2 demonstrates how these micromotors move by both bubble push ([Supplementary-material supplementary-material-1]) and fluid jet ([Supplementary-material supplementary-material-1]) mechanisms at short time scales. In case of an axisymmetric micromotor (including perfectly spherical Mg particle, no shell defects, and an axisymmetric circular opening), a Mg-TiO_2_ micromotor and the ejected bubbles move rectilinearly along the symmetry axis. Figure 2(a) shows an experimental realization of an axisymmetric micromotor with an almost uniform distribution of bubble size. Upon formation and growth, each bubble pushes the micromotor forward by exerting force on the micromotor and the bubbles in the tail. Such bubble-push process is the main mechanism during the initial stages of the micromotor lifetime. Thus, as shown in Figure 2(a), iii, the micromotor's net displacement (the distance from micromotor's initial position at *t* = 0) consists of discrete steps. Since there is no significant backward motion upon bubble ejection, the length (solid red line) of the micromotor travel path almost coincides with the net displacement (dashed blue line).

The structural and dynamic symmetries have a significant influence on the operation of micromotors. While structural asymmetries [13, 32] appear in typical transient micromotors (Figure 2(b)) as a result of imperfections in materials and fabrication process, dynamic asymmetries can occur during the operation of both axisymmetric and typical micromotors. The structural asymmetries in a typical micromotor may result from nonisotropic Mg particles, defects and nonuniformity in the shell, or asymmetry in the opening as artifacts of the fabrication process (Figure 2(b), ii). On the other hand, the dynamical asymmetries appear as a result of random nucleation of bubbles with different sizes at several locations inside the micromotor (after significant depletion of the Mg core) and their interactions, coupled with their ejection at various angles or extended growth while attached to the micromotor. Additionally, environmental noise, fluid convection, and buoyancy force affect the motion of these micromotor. Thus, the length of the micromotor trajectory will differ greatly from its displacement. (Figure 2(b), iii). The asymmetry effects become more pronounced once a portion of the Mg core is depleted and a cavity is formed inside the micromotor. The bubbles can nucleate and grow at different locations inside the cavity before being ejected. Upon the sudden formation of each bubble inside the cavity, a corresponding volume of the fluid is ejected out from the opening. This sudden fluid jet results in hopping of the micromotor (Figure 2(c) and [Supplementary-material supplementary-material-1]). The intensity of the fluid jet and the hopping distance depends on the bubble size inside the cavity and the size of the micromotor's opening. As illustrated in Figure 2(c) upon bubble nucleation inside the cavity, the micromotor hops forward, the bubble is ejected joining to the train of bubbles, and the micromotor is stationary until the next bubble is nucleated inside the cavity.

The majority of the micromotors behave similarly to the typical micromotor shown in Figure 2(b). There are usually structural and dynamic asymmetries involved whose effect may vary over the lifetime of the micromotor, and sequences of bubbles with random sizes and ejection times are formed. To find order in this complex system, we aimed to identify the elements of motion upon which the dynamics of a micromotor is built. We looked closely at how bubbles form and where the bubble size variation comes from. In all of the experiments, we used a solution containing a surfactant (0.2% of Triton X-100) to stabilize the bubbles; yet, it is useful to examine how different amounts of surfactant will change the overall bubble size. The dependence of the bubble size upon the surfactant concentration is presented in [Supplementary-material supplementary-material-1]. As expected, higher surfactant concentration resulted in smaller bubble diameter while averaging over all gears. To build a framework for quantitative analysis, we categorized the bubble formation mechanism into three bubble ejection processes which contribute to the propulsion differently. Thus, in analogy with a macroscopic multigear machines, we call each process a *gear*.

**Figure 3:**
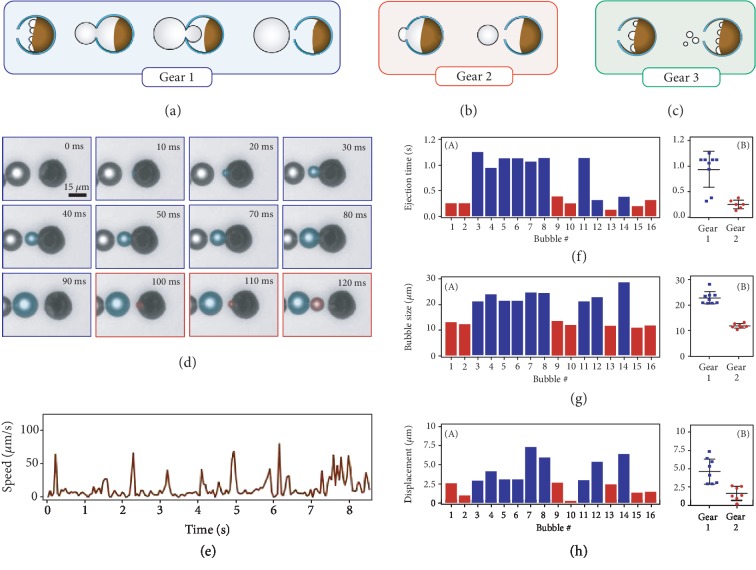
Three gears of bubble growth and ejection. (a) Gear 1 features a long growth period outside the motor and large bubble size. (b) Gear 2 features a short growth period inside the motor and medium bubble size. (c) Gear 3 features a negligible growth period and immediate ejection of small bubbles. (d) Time lapse of Mg-TiO_2_ micromotor demonstrating Gears 1 and 2 for two consecutive bubbles. (e) Speed of the micromotor from (d) over a longer (8.5 s) span. (f) (A) Time to eject a bubble for the 8.5 s span of the micromotor from (d) providing distinction between Gear 1 (blue) and Gear 2 (red). (B) Clustering of bubble ejection time data with the mean and standard deviation. (g) (A) Bubble size for the 8.5 s span of the micromotor from (d) providing distinction between Gear 1 (blue) and Gear 2 (red). (B) Clustering of bubble size data with the mean and standard deviation. (h) (A) Motor displacement for the 8.5 s span of the micromotor from (d), providing distinction between Gear 1 (blue) and Gear 2 (red). (B) Clustering of motor displacement data with the mean and standard deviation.

At the early stages of micromotor operation, bubbles nucleate on the Mg surface and grow on the micromotor surface before detachment. At later times, upon cavity formation inside the micromotor, some bubbles grow first inside the cavity and then continue to grow while a portion of them is outside of the micromotor (Figure 3(a)). We call this process “Gear 1.” Some of the bubbles only grow inside the cavity and suddenly hop out of the micromotor. We call these “Gear 2” (Figure 3(b)). Due to the extended growth period, on average a Gear 1 bubble grows to a larger size than a Gear 2 bubble. We also observed the ejection of very small bubble, which usually does not contribute strongly to propulsion (Figure 3(c)). We call this small bubble ejection Gear 3. This gear can result from many nucleation events taking place at the same time inside the cavity, forcing some small bubbles to hop out before having enough time to grow. In summary, Gear 2 is more prevalent in the early stage of a micromotor's life while all gears are prevalent in the middle of the lifetime as the cavity inside has expanded with depleted Mg. Finally, at the end, we see sporadic Gears 1 and 3 bubbles before the motion stops.

To illustrate the differences between Gear 1 and Gear 2, we present in Figure 3(d) a micromotor showcasing both modes of bubble production ([Supplementary-material supplementary-material-1]). During the first 10 ms of the time lapse, we see the nucleation of a bubble (highlighted in blue). By the 20 ms mark, the bubble is already outside the motor but has not detached and is continuing to grow until about 80 ms at which point it has grown to a size comparable to the size of the micromotor. Finally, the bubble detaches, and the micromotor moves forward. The displacement between the bubble and micromotor at 90 ms is due to the fluid jet caused by the nucleation of other bubbles inside the micromotor. The growth phase for the next bubbles (Gear 2, highlighted by red) is much shorter (30 ms). The bubble nucleates and grows inside the micromotor structure and is ejected out. While the bubbles of Gear 1 have time to grow on the surface, the Gear 2 bubbles are limited in size to the space inside the cavity and are thus expected to be smaller.

We analyzed the micromotor in Figure 3(d) for a longer period of 8.5 s to quantify the behavior of Gear 1 and Gear 2 bubbles and their contribution to motion. The micromotor did not eject a Gear 3 bubble during the time interval of our analysis. We observed that the sequence of gear occurrence is random which we attribute to the stochastic processes involved in bubble nucleation and ejection. Therefore, we statistically quantified the significance of each gear. The micromotor speed varies over time (Figure 3(e)). There are periods of inactivity in displacement mostly during the growth phases of Gear 1 bubbles followed by large spikes in speed due to the large displacement from bubble push or fluid jetting. To differentiate the behavior of the gears quantitatively, for each bubble, we extracted the time required to eject the bubble, the bubble size, and the micromotor displacement due to each bubble formation and ejection. The time it takes to eject a bubble from the micromotor (Figure 3(f)) ranges around 0.2 ± 0.06 s for Gear 2 bubbles while Gear 1 bubbles take more than three times longer with an average of 0.64 ± 0.34 s. A similar trend is observed in the bubble size (Figure 3(g)). Gear 2 bubbles are smaller with an average size of 12.1 ± 0.85*μ*m while Gear 1 bubbles grow up to 20.3 ± 7.25*μ*m. Finally, Gear 2 bubbles result in a smaller displacement of 1.6 ± 0.86*μ*m (Figure 3(h)) compared to Gear 1 bubbles with average displacement of 4 ± 1.96*μ*m.

**Figure 4:**
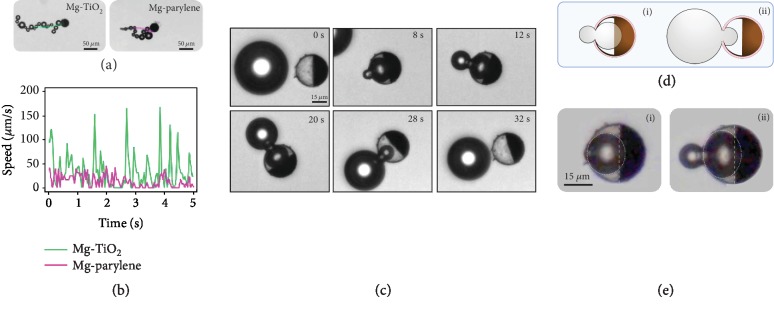
Effect of shell material on micromotor behavior. (a) Motion of Mg-TiO_2_ and Mg-parylene micromotors and (b) their corresponding speeds. (c) In the mid-lifetime of the Mg-parylene micromotor, the time required for Gear 1 bubble ejection is very slow (more than 30 times that of a Mg-TiO_2_ micromotor) and the generated bubbles are typically larger than the micromotor. (d) The Mg surface inside the micromotor is concave (i) and the bubble have more space to grow, thus (ii) the ejected bubble can grow larger than the motor size. (e) Circle fitting to a bubble (i) fully inside and (ii) partly outside the micromotor.

Having established a multigear dynamics framework, an important design question may arise: can we engineer the micromotor structure such that we can have more control over the bubble propulsion process and the occurrence of gears with fine-tuned bubble properties? A comprehensive answer to this question requires exploring the design parameter space of the micromotor with different material properties and symmetry considerations, and this will be the scope of our future work. However, within the scope of the current study, we demonstrate qualitatively the effect of material selection on multigear bubble propulsion. The presented results so far have been based on a hydrophilic TiO_2_ shell. Changing the shell to hydrophobic parylene significantly affects the bubble nucleation, growth, and ejection, and thus the overall micromotor propulsion (as shown in Figure 4(a) and [Supplementary-material supplementary-material-1]).

During the first one minute of a micromotor's lifetime, a typical Mg-TiO_2_ micromotor (Figure 4(a)) shows higher average speed and displacement than a typical Mg-parylene micromotor. A micromotor with a parylene shell has a lower bubble generation frequency than a Mg-TiO_2_ one and longer bubble growth periods, both accompanied by diminished displacement over time. The speed spikes of an Mg-parylene micromotor are much smaller than that of a Mg-TiO_2_ micromotor (Figure 4(b)). Figure 2(c) shows the formation of a bubble by an Mg-parylene micromotor halfway through its lifetime. The bubble grows in the middle, part of it extends out of the opening, whereby it continues to grow for a very long time until finally detaching from the micromotor ([Supplementary-material supplementary-material-1]). This is a clear demonstration of Gear 1 and here the bubble grows up to a size larger than the micromotor itself (Figure 4(c)). The time of Gear 1 bubble growth and ejection for a micromotor with hydrophobic shell is more than 30 times longer than compared to a micromotor with a hydrophilic shell (Figure 3(f), B).

Halfway through the micromotor lifetime, while the majority of bubbles for hydrophobic shell micromotors are Gear 1, hydrophilic shell micromotors produce a random train of Gears 1, 2, and 3 at a much higher rate and smaller bubble size. We observed a major structural distinction in the curvature of Mg inside a micromotor after the formation of the cavity. As shown in [Supplementary-material supplementary-material-1], for a hydrophilic shell micromotor, the Mg core becomes slightly convex in shape, with the Mg core being dissolved most rapidly at the edges. We speculate that as gastric acid enters the micromotor, it preferentially wets the sides of the micromotor, thus consuming faster the Mg at the sides. Additionally, it is easier to nucleate a bubble on the side adjacent to a TiO_2_ wall as opposed to the middle of the Mg core. Conversely, as schematically presented in Figure 4(d), i, it is our understanding that the Mg surface in a hydrophobic shell micromotor is concave. While we do not have a way to observe the curvature inside the micromotor directly, we deduced from the circle fitting and the angle between the bubble and the equator line created by Mg that the metal surface is concave (Figure 4(e)). As such, the bubble has space to grow to a larger size inside the cavity which supports and keeps the part of the bubble inside the cavity while a portion of the bubble grows on the micromotor surface. As a result, the outside part has more time to grow before the surface tension at the water-bubble interface, the pressure inside the bubble, and the curvature of the bubble around the opening pull the inside part out (Figure 4(d), ii).

**Figure 5:**
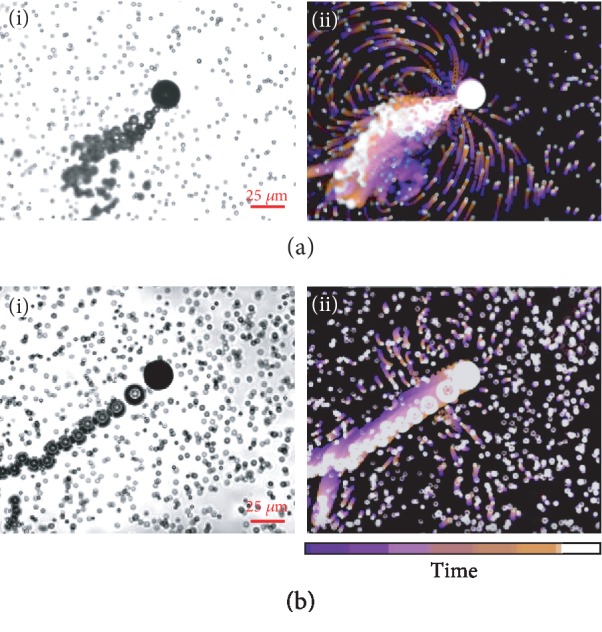
Fluid flow around Mg-TiO_2_ micromotors. The flow field generated by a (a) stationary and (b) motile Mg-TiO_2_ microengine. (i) is an instant of micromotor operation and (ii) illustrates the overlaps [34] of a stack of 50 fluorescent microscopy images (during a 4 sec period) to show the flow field around the micromotor.

The effect of gear type and occurrence frequency on bubble propulsion, at more fundamental level, is manifested in the pattern of the reactive fluid flow inside and around the micromotor. A detailed analysis of the effects of gears on fluid dynamics is beyond the scope of our current study. Here, we provide general discussion on the pattern of fluid flow generated by bubble production mechanism.  Figure 5 and [Supplementary-material supplementary-material-1] demonstrate the operation of a stationary and a motile Mg-TiO_2_ microengine. The bubble ejection of a stationary microengine near a substrate induces a pattern of circular flow (Figure 5(a)) similar in shape but opposite in direction to a puller microorganism under confinement [33]. These filter feeder microorganisms mix their local environment by circular flow and bring food to their mouth. The similar biomimetic flow pattern of transient micromotors can serve the same purpose by enhancing the transport of reactive materials in the fluid toward the opening of the microengine which provides efficient local mixing. The biomimetic flow pattern changes when the stationary microengine turns into a motile micromotor. Instead of circular flow, the translocation of freely moving microengines generates disordered open streamlines. (Figure 5(b)). The remarkably enhanced fluid dynamic resulting from the transient micromotor platform offers considerable promise for increasing the efficiency of a variety of medical and decontamination processes.

## 3. Conclusion

In conclusion, we have established a multigear bubble propulsion framework to analyze the stochastic sequence of bubbles generated during the motion of transient micromotors. We identified three modes of bubble ejection. The bubbles that continue to grow outside the micromotor tend to be larger and take more time for ejection but present larger micromotor displacement. We investigated the distinct effects of hydrophobic and hydrophilic shell on the operation of micromotors including bubble nucleation rate, bubble size, and the bubble ejection processes. A more detailed investigation of the effect of material selection upon multigear dynamics and discovering design principles for fine tuning the dynamics of multigear bubble propulsion micromotors will be the subject of our future studies. Our analysis of the fluid flow pattern around the microengines and its similarities to flow around filter feeder microorganisms suggest new possibilities aligned with our previous studies [33] for exploiting the transient micromotors for enhanced local mixing and environmental remediation. Our analysis framework provides a pragmatic guideline to quantitatively study the complex system of multigear bubble propulsion and investigate the effect of the material and environmental parameters (including additional parameters involved in other biological media such as serum or interstitial fluid) on elements of motion. Our analysis framework provides new insights and understanding of the bubble propulsion of chemical micromotors and can be extended to other transient micromotor structures where multiple bubble production mechanisms may change with time. Our study guides future design principles of transient micromotors and serves for fine tuning the performance of these micromotors.

## 4. Materials and Methods

### 4.1. Micromotor Fabrication

The Mg-based micromotors were prepared using commercially available magnesium (Mg) microparticles (catalog #FMW20, TangShan WeiHao Magnesium Powder Co.; average size, 20 ± 5*μ*m) as the core. The Mg microparticles were initially washed with acetone to eliminate the presence of impurities. After drying under a N_2_ current, the Mg microparticles were dispersed onto glass slides (10 mg of Mg microparticles per glass slide) and coated with TiO_2_ by atomic layer deposition (ALD) (at 100°C for 3000 cycles) using a Beneq TFS 200 system. Being a chemical vapor deposition technique, ALD utilizes gas phase reactants, leading to uniform coatings over the Mg microparticles, whereas still leaving a small opening (~2 *μ*m) at the contact point of the microparticle to the glass slide. Mg-parylene micromotors were prepared in a similar fashion as Mg-TiO_2_ micromotors. After dispersion of Mg particles onto glass slides, a parylene coating was deposited via a PDS 2010 Labcoter 2 Parylene deposition system. The samples were rotated during the deposition and the chamber was kept at 135°C. The coating thickness is estimated at ~500 nm.

### 4.2. Micromotor Propulsion

The simulated gastric fluid was obtained by diluting concentrated simulated intestinal fluid (Sigma-Aldrich, 14666) 10 times and supplementing with 0.2% Triton X-100 (Fisher Scientific, 9002-93-1) as a surfactant. The autonomous propulsion of the Mg-based micromotor happened in the simulated gastric fluid. An inverted optical microscope (Nikon Eclipse Ti-S/L100) coupled with different microscope objectives (10x, 20x, and 40x) along with a Hamamatsu C11440 digital camera and NIS Element AR 3.2 software was used to capture the videos. Additionally, the speed of the micromotors was tracked using a NIS elements tracking module and the flowtrace ImageJ plugin.

### 4.3. Micromotor Characterization

SEM images of Mg-based micromotors were obtained with an FEI Quanta 250 ESEM instrument, using an acceleration voltage of 10 kV. EDX mapping analysis was performed using an Oxford EDX detector attached to SEM instrument and operated by Pathfinder software.

## Supplementary Materials

Supplementary Materials Figure S1: fabrication of transient micromotor. A monolayer of Mg particles are positions on a glass slide and a layer of insulator (TiO_2_ or parylene) is deposited on the Mg particles using atomic layer deposition technique. Figure S2: alternative representation of Figure 1(e) with both data and fit as lines. Figure S3: bubble size variation with surfactant concentration. (a) Bubble size normalized by the size of the micromotor as a function of surfactant Triton X-100. (b) Images of typical micromotors and their bubble tails for (i) 0%, (ii) 0.05%, and (iii) 0.5% Triton X-100. Figure S4: the bubble formation inside a micromotor with a hydrophilic shell. Supporting Video Descriptions Supporting Video S1: axisymmetric and typical propulsion of Mg micromotors. Supporting Video S2: micromotor propulsion by sudden fluid jet mechanism. Supporting Video S3: mechanisms of bubble growth and ejection. Supporting Video S4: effect of shell material on micromotor behavior. Supporting Video S5: fluid flow around Mg-TiO_2_ microengines.
