# Supplementary material for: Multigear Bubble Propulsion of Transient Micromotors
Source: Research (Wash D C). 2020 Feb 21;2020:7823615. doi: 10.34133/2020/7823615 (PMC7054719; doi:10.34133/2020/7823615)
Supplement: Supplementary Materials — Figure S1: fabrication of transient micromotor. A monolayer of Mg particles are positions on a glass slide and a layer of insulator (TiO2 or parylene) is deposited on the Mg particles using atomic layer deposition technique. Figure S2: alternative representation of Figure 1(e) with both data and fit as lines. Figure S3: bubble size variation with surfactant concentration. (a) Bubble size normalized by the size of the micromotor as a function of surfactant Triton X-100. (b) Images of typical micromotors and their bubble tails for (i) 0%, (ii) 0.05%, and (iii) 0.5% Triton X-100. Figure S4: the bubble formation inside a micromotor with a hydrophilic shell. Supporting Video Descriptions Supporting Video S1: axisymmetric and typical propulsion of Mg micromotors. Supporting Video S2: micromotor propulsion by sudden fluid jet mechanism. Supporting Video S3: mechanisms of bubble growth and ejection. Supporting Video S4: effect of shell material on micromotor behavior. Supporting Video S5: fluid flow around Mg-TiO2 microengines. [file 7823615.f1.zip › SI-StochasticMultiGear-Clean 1-6-2020.docx]

**SUPPORTING INFORMATION**

**Multi-gear bubble propulsion of transient micromotors**

*Amir Nourhani,^1,2^*^†^ *Emil Karshalev,^1^ Fernando Soto,^1^ Joseph Wang^1,^*^†^

1 Department of NanoEngineering, University of California San Diego, La Jolla, CA 92093, USA.

2 Departments of Mechanical Engineering and Biology, University of Akron, Akron, OH 44325, USA.

† Corresponding authors.

Email: [josephwang@eng.uscd.edu](mailto:josephwang@eng.uscd.edu)
 nourhani@uakron.edu

**Supporting Figure**


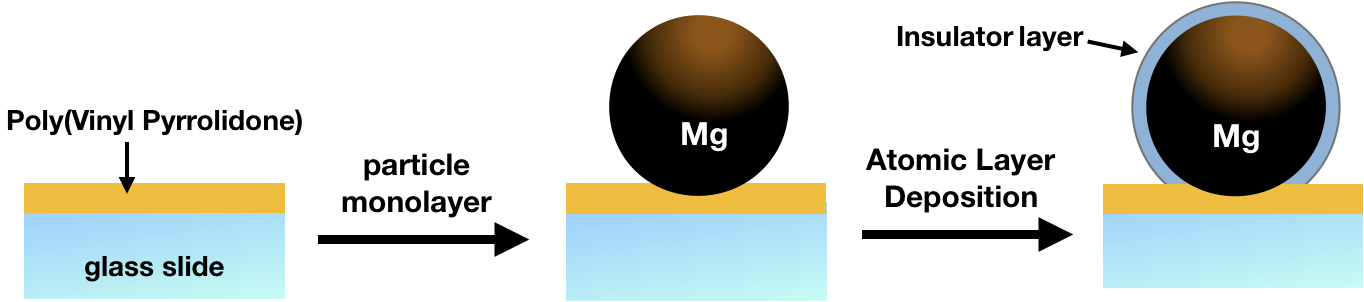


**Fig. S1**. **Fabrication of transient micromotor**. A monolayer of Mg particles are positions on a glass slide and a layer of insulator (TiO_2_ or parylene) is deposited on the Mg particles using atomic layer deposition technique.


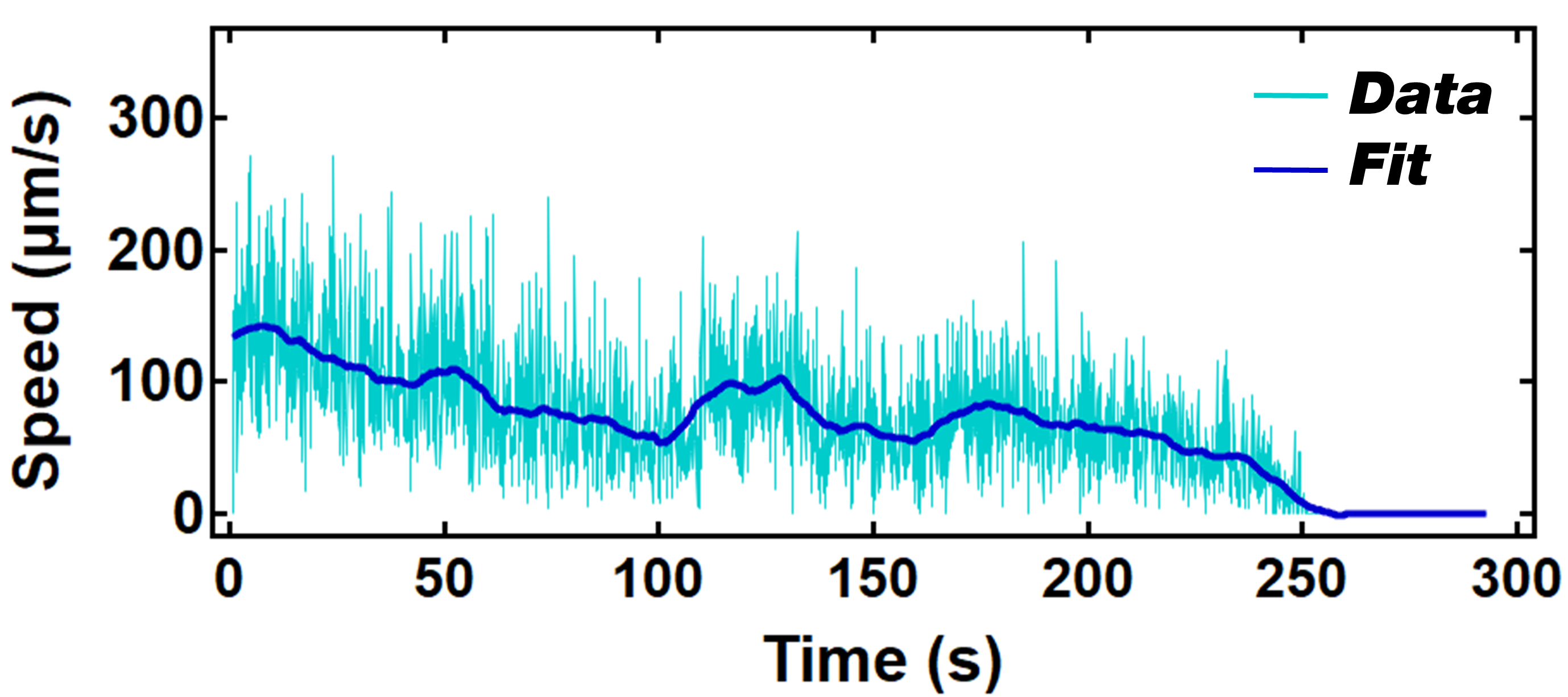


**Fig. S2. Alternative representation of Fig. 1e with both data and fit as lines.**


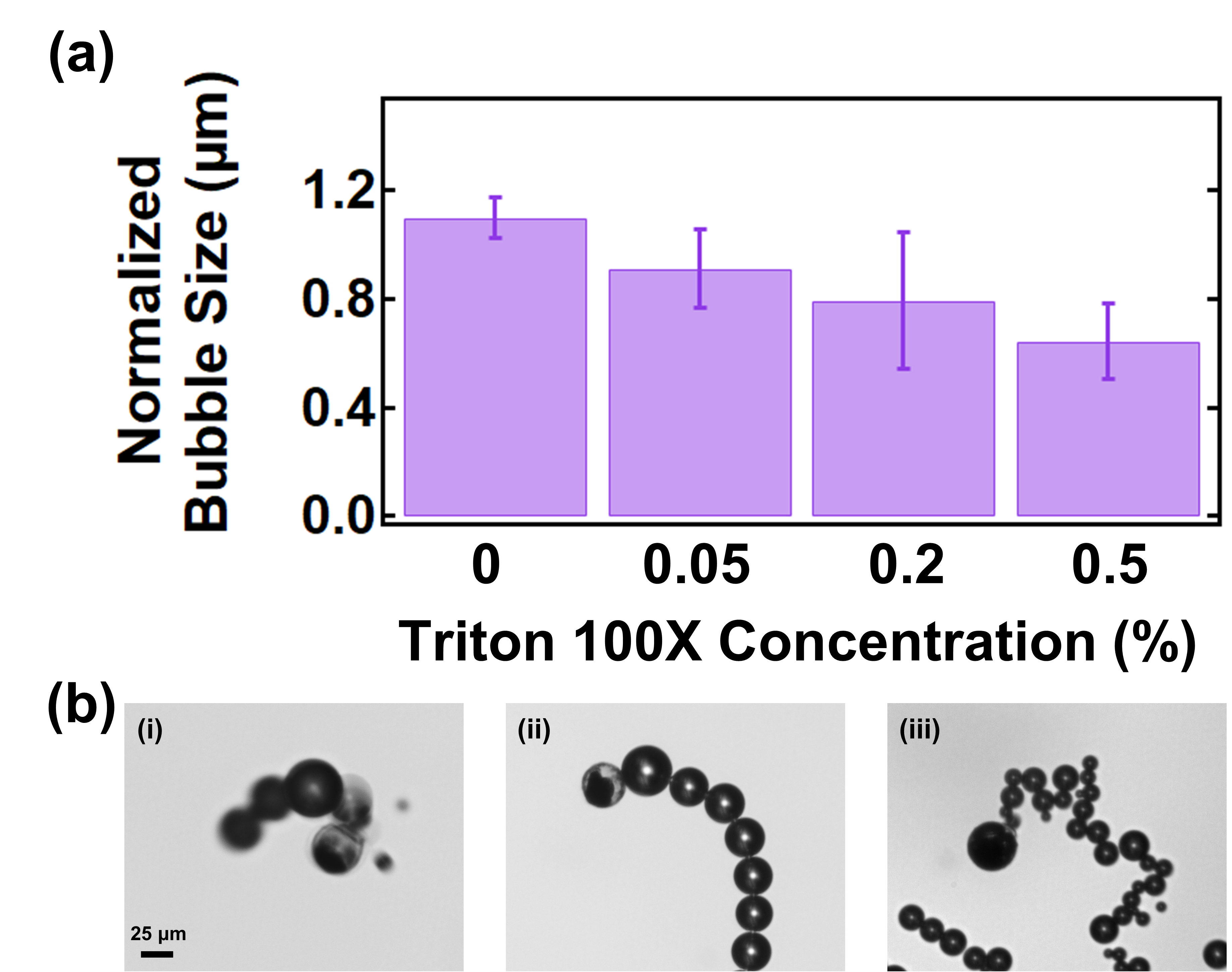


**Fig. S3. Bubble size variation with surfactant concentration.** (a) Bubble size normalized by the size of the micromotor as a function of surfactant Triton X-100. (b) Images of typical micromotors and their bubble tails for (i) 0%, (ii) 0.05%, and (iii) 0.5% Triton X-100


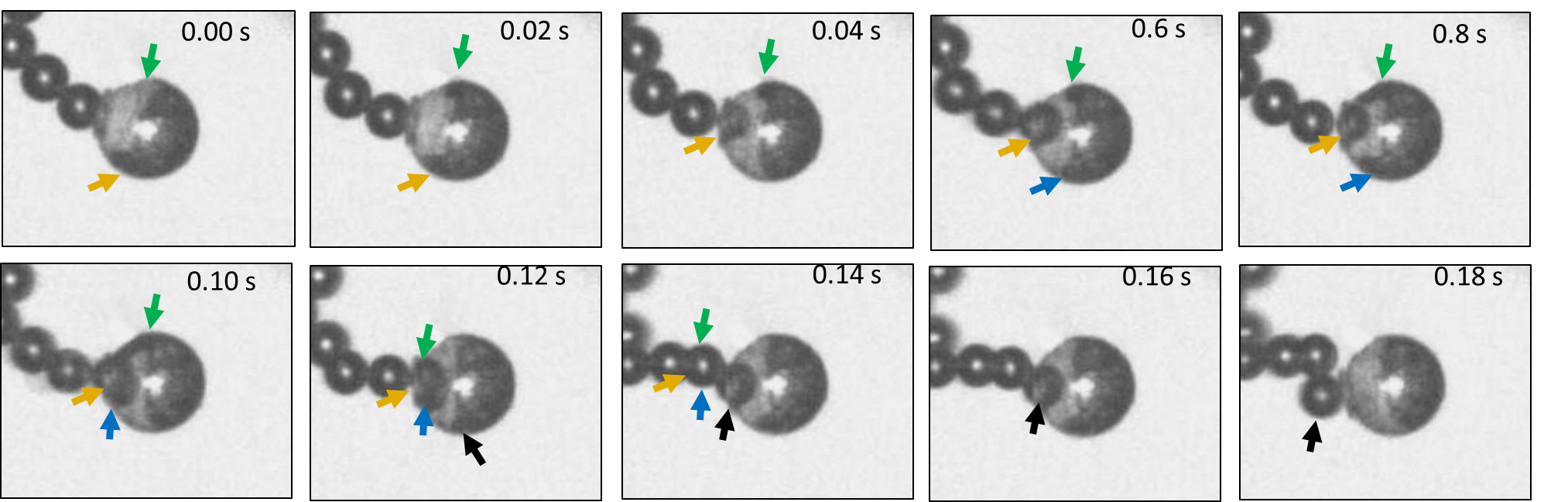


**Fig. S4. The bubble formation inside a micromotor with a hydrophilic shell.**

**Supporting Video Descriptions**

**Supporting Video S1.** Axisymmetric and typical propulsion of Mg micromotors.

**Supporting Video S2.** Micromotor propulsion by sudden fluid jet mechanism.

**Supporting Video S3.** Mechanisms of bubble growth and ejection.

**Supporting Video S4.** Effect of shell material on micromotor behavior.

**Supporting Video S5.** Fluid flow around Mg-TiO_2_ microengines.
